# Psychological Considerations in Pediatric Chronic Illness: Case Examples

**DOI:** 10.3390/ijerph17051644

**Published:** 2020-03-03

**Authors:** Jennifer M. Rohan, Tanvi Verma

**Affiliations:** 1 Children’s Hospital of Richmond at Virginia Commonwealth University, Richmond, VA 23219, USA; tverma@vcu.edu; 2 Cancer Prevention and Control Program, Massey Cancer Center, Virginia Commonwealth University, Richmond, VA 23219, USA; 3 Virginia Commonwealth University School of Medicine, Richmond, VA 23219, USA

**Keywords:** adherence, prevention/control, health behavior, psychology, pediatrics

## Abstract

Despite significant gains in survival rates for pediatric patients and adolescents/young adults (AYA) with chronic illness, patients in this vulnerable age group are also at an increased risk for developing one or more adverse effects related to their disease, treatment, or maladaptive health behaviors. Maladaptive health behaviors ultimately increase the risk for developing adverse effects, including: increased rates of morbidity and mortality, impaired physical functioning, increased fatigue, obesity, increased psychological distress, and poor quality of life. With close attention including participation in preventive and therapeutic health promotion interventions, problematic health behaviors can be mitigated and ultimately prevented over time. It is well known that improved psychological functioning and adaptive coping can result in improved health status. The present paper provides four case examples illustrating various psychological interventions in pediatric chronic illness. As evidenced in the four case examples, pediatric psychologists provide comprehensive interventions for patients with acute and chronic medical conditions through the use of health promotion interventions, adherence and self-management promotion, cognitive behavioral therapy, behavioral therapy, medical coping, parent training, and motivational interviewing. Our case series demonstrates that for the most impactful behavior change to occur, a combination of interventions is often the most effective.

Despite significant gains in survival rates for pediatric patients and adolescents/young adults (AYA) with chronic illness, patients in this vulnerable age group are at an increased risk for developing one or more adverse effects related to their disease, treatment, or maladaptive health behaviors. Maladaptive health behaviors can ultimately increase the risk for developing adverse effects, including: increased rates of morbidity and mortality, depression, self-harm and suicide, body esteem issues, impaired physical functioning, increased fatigue, obesity, increased psychological distress, and poor quality of life [1,2,3,4,5,6,7]. Although not a focus of the present paper, high-risk behaviors like sexual activity and substance abuse are also common among adolescents and young adults, which could ultimately impact health behaviors and outcomes. Thus, pediatric and adolescent/young adult (AYA) patients with chronic illness should not be excluded from assessment of risky health behaviors like sexual activity and substance abuse. Valencia et al. [8] found that adolescents with chronic illness often report decreased knowledge and use of contraceptives. However, adolescents with chronic illness reported lower rates of substance use and other delinquent behaviors compared to their healthy peers, with alcohol being reported as the most frequent substance used [8]. It is well documented that there is individual variability in health behaviors across the illness trajectory, especially within adolescent and young adult patient populations. With close attention including participation in preventive and therapeutic health promotion interventions, problematic health behaviors can be mitigated and ultimately prevented over time.

Tailored and personalized preventive and therapeutic interventions targeting specific risk and protective factors that may impact health behaviors and ultimately health outcomes for individual patients are very much needed to reduce the risk for adverse effects [9,10,11]. There is strong evidence that patients diagnosed with chronic illness often report increased psychological distress including higher levels of anxiety and depression, decreased physical activity, increased fatigue, and decreased quality of life [12,13,14]. Chronic diseases common in adolescents and young adults (AYA), such as asthma, are associated with biological markers (e.g., neuropeptide Y), which are associated with psychological stress [15,16]. In a study investigating psychological needs of AYA cancer survivors, 82% of survivors expressed having at least one concern related to behavioral, cognitive, and emotional domains; however, only 38% of these patients were referred for, and received, psychological treatment [17]. Furthermore, pediatric patients suffering from leukemia also demonstrated low physical, social, and academic functioning [9,18,19,20]. It is clearly evident that all patient populations would benefit from receipt of psychological services [13,19].

It is well known that a patient’s health beliefs (i.e., how one views their own illness and associated treatment) will likely influence health behaviors [9,18,19]. Those patients who perceive their illness as serious, perceive themselves as vulnerable, and recognize the importance of engaging in healthy behaviors across the illness trajectory, are more likely to engage in positive health behaviors over time; these include participating in preventive and therapeutic interventions focused on psychological health and well-being, engaging in physical activity, and maintaining healthy lifestyle patterns like optimal adherence, good sleep hygiene, and healthy diets [9,18,19]. 

Improved psychological functioning and adaptive coping can result in improved health status [21]. Although pediatric and AYA patients both report increased psychological distress, adjustment difficulties, and decreased quality of life due to their chronic illness and treatment-related burden, AYA patients often report additional difficulties specific to this challenging developmental period; these include a threatened sense of safety and reduced security, feelings of loss of control, body image concerns, decreased self-esteem and modified sense of self, difficulties with interpersonal relationships, disruptions in daily life (e.g., academics, employment), increased parent–adolescent conflict, and a threatened sense of independence [22]. 

## 1. Case Examples

The cases and corresponding data that are described in the present paper were exempt from obtaining Institutional Review Board (IRB) approval. IRB approval was only required for case studies/series that involved more than five patients. All patients and families consented to treatment and provided verbal assent (<18 years old) or verbal consent (>18 years old) for use of their de-identified information.

### 1.1. Case 1. Psychoeducation Regarding the Importance of Preventative Mediations for Pediatric Asthma

The first patient whom we will discuss is a 14-year-old African American male diagnosed with severe, persistent asthma. He was referred to a pediatric psychologist for a history of chronic nonadherence to oral steroids and inhaled corticosteroids, parent–child conflict, and maladaptive health behaviors impacting his health. Prior to the referral, the patient had one emergency visit for an asthma exacerbation, which was preceded by a prolonged hospitalization including the need for intubation and receipt of care in the intensive care unit (ICU). The pulmonary team suspected that his recent emergency room visits and prolonged hospitalization were likely related to medication nonadherence in the setting of increased parent–adolescent conflict. The patient lived with his biological maternal grandmother who was his primary caregiver and legal guardian. He was the oldest of five children all of whom were living in the same household. The family had a number of psychosocial stressors including multiple family members receiving care for chronic illnesses, financial difficulties and low socioeconomic status, and ongoing housing issues, including having mold and roaches, which can be triggers for asthma symptoms.

**Figure 1:**
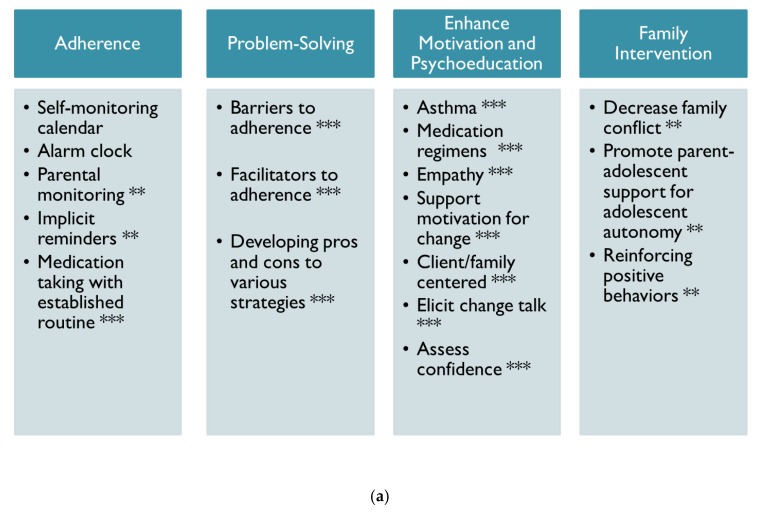

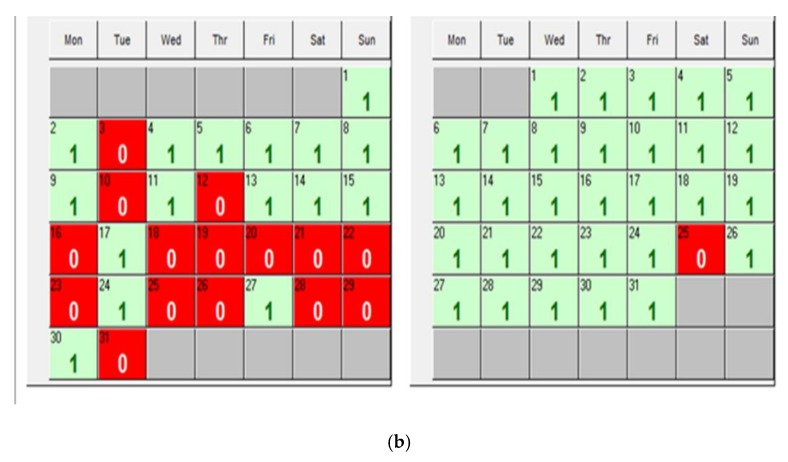
(**a**) Treatment strategies for chronic nonadherence, health behaviors, and improving family dynamics (Case 1). Note: Strategies with ** represent intermittent improvements in medication adherence for short periods of time and *** represents prolonged periods of improved medication adherence. (**b**) Example of results obtained from electronic monitoring of medication adherence (Case 1).

As shown in Figure 1a, psychological treatment included a multi-faceted approach, which included adherence and health promotion interventions, problem-solving, motivational interviewing, and family-focused interventions. This patient also received an electronic monitor to assess objective patterns of medication adherence to oral steroids. The primary focus with this patient was improving his medication adherence by identifying barriers to treatment success. This patient and his grandmother also received consistent feedback regarding his medication adherence (see Figure 1b) and family-centered interventions. During each session, the electronic monitoring device was downloaded, and medication adherence results were reviewed to identify barriers to success. One factor contributing to poor adherence was forgetting to take medications, thus multiple strategies were used to identify which specific set of strategies improved his medication adherence (e.g., self-monitoring calendar, alarm clock, parental monitoring, implicit reminders like pairing medication with established routines, post-it notes, etc.). 

This patient also reported that he was not aware of the rationale for taking preventative daily oral medications for asthma. Thus, part of our sessions was focused on providing education about asthma and medication regimens. Motivational interviewing was used to identify patient-centered factors motivating health behavior change. Finally, the patient and grandmother engaged in family interventions to decrease parent–child conflict and promote parent support for adolescent autonomy. As shown in Figure 1b, the patient’s medication adherence improved over the course of one month after receipt of weekly to every other week psychological interventions. 

### 1.2. Case 2. When the Future is So Far Away: Unique Challenges for AYA Patients

As reviewed above and demonstrated in the previous case, AYA patients with a chronic illness often report unique challenges when managing their chronic illness in the setting of a critical developmental period. A 19-year-old African American female diagnosed with sickle cell disease SS-type was referred to a pediatric psychologist for a chronic history of nonadherence to medication. The patient reported that she had not taken her medication as prescribed in over five years. At the time of referral, she reported an increase in sickle cell pain crises and reluctance to take medication for her disease or for pain. It is notable that she had a history of silent and sub-clinical strokes related to her sickle cell disease. Based on prior neuropsychological testing, results indicated a borderline-to-low average IQ with significant variability across subscales. She also was diagnosed with a specific learning disability in reading and deficits in attention and executive functioning. Despite these deficits, this young woman was completely responsible for managing her own medical care, which is a strength; however, she often refused to receive help from her mother or other support partners in her life even when needed. 

**Figure 2:**
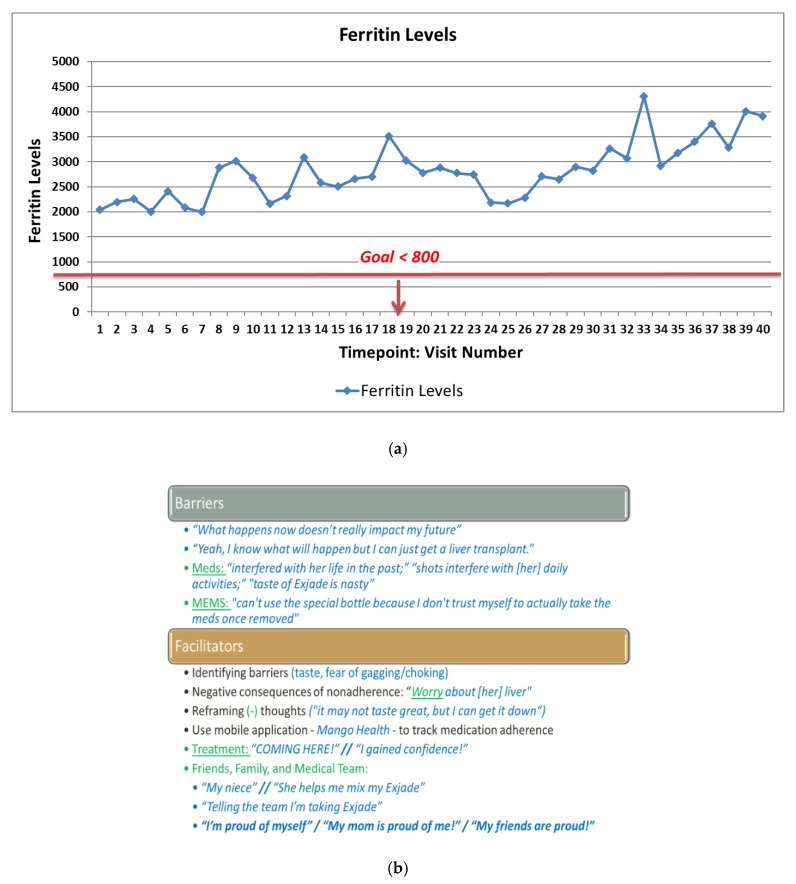
(**a**) Ferritin levels for a sickle cell patient with a history of chronic nonadherence (Case 2). Note: Higher ferritin levels indicate worse disease management. Ferritin values <800 indicate better disease management and ultimately result in better health outcomes. (**b**) Barriers and facilitators to adaptive health behaviors in sickle cell disease (Case 2).

When meeting the psychologist for the initial session, she presented significant reluctance around working with a pediatric psychologist indicating that she had seen several psychologists over the past five years and reported low confidence that this psychologist would enhance her motivation to engage in healthy lifestyle behaviors. As shown in Figure 2a, this specific patient had a chronic history of poor disease management over a course of five years as evidenced by significantly higher ferritin levels than what is recommended for pediatric sickle cell patients. The amount of ferritin stored reflects the amount of iron stored; at that time, good disease management was indicated by levels <800. As shown in Figure 2a, this specific patient had lab values much higher than what was recommended and thus was at an increased risk for adverse effects, including liver failure. 

As shown in Figure 2b, a number of intervention strategies were effective with improving this patient’s self-management behaviors, adherence, and health behaviors. When beginning treatment, this patient frequently reported that her future was so far away, thus she was not initially motivated to make adaptive behavior changes. The first several sessions were focused on patient-centered strategies for enhancing motivation for behavior change, including providing psychoeducation around the relationship between poor self-management during childhood and adolescence and the relationship to future health outcomes. We also spent several sessions on identifying barriers to taking medication, which revealed a significant fear of choking on medications due to the texture of the medication. This allowed the clinician to focus on strategies for reducing these fears, which ultimately improved her medication adherence over time. What was most interesting was that this patient indicated she would not be a good candidate for using an electronic monitoring device to measure adherence over time due to a fear that she would open it and not take the medication. Thus, we identified an alternative tracking system (MangoHealth), which she reported that she would use “honestly” because it provides her with reminders to take her medication with the option of saying she did not take it versus taking it later. MangoHealth demonstrated an increase in adherence rates over a period of three weeks from 0% to 70%. This patient reported that medication adherence improved as a result of increased support from her family and friends, including receiving positive feedback from friends and family on Facebook. She also involved her younger niece in helping her mix her medication, which allowed her to recognize the importance of medication adherence in the present for ensuring she would be able to spend time with her niece as she gets older. Finally, identifying her fears associated with medication taking, decreasing her anxiety, and providing her with anticipatory guidance contributed to patterns of adaptive health behavior change.

### 1.3. Case 3. Medication Adherence in Young Children

While adolescents and young adults may be primarily responsible for managing their chronic illness, it also is important for young children to be active participants in their illness management. This case provides an illustration for how medication adherence can also be a challenge for younger children. A 5-year-old white male diagnosed with acute lymphoblastic leukemia was presented to psychology due to increased resistance with taking oral medication (“medication taking was a fight” per his parents). Parents reported that he was likely having a harder time now due to new onset pill swallowing difficulties in the setting of complaining about the taste and texture of oral medications. This young man was prescribed liquid 6-mercaptopurine, which was given daily, and liquid methotrexate, which was given for a short period of time every month. He lived with his mother and father and was an only child.

**Figure 3:**
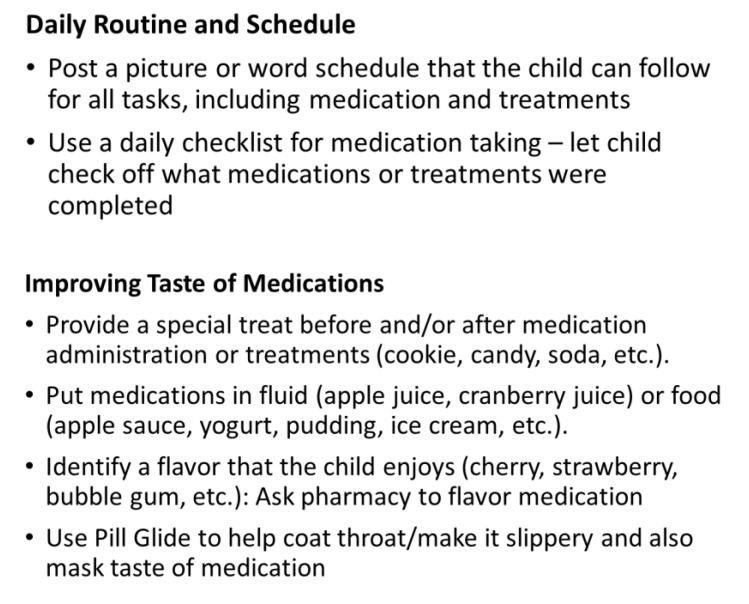

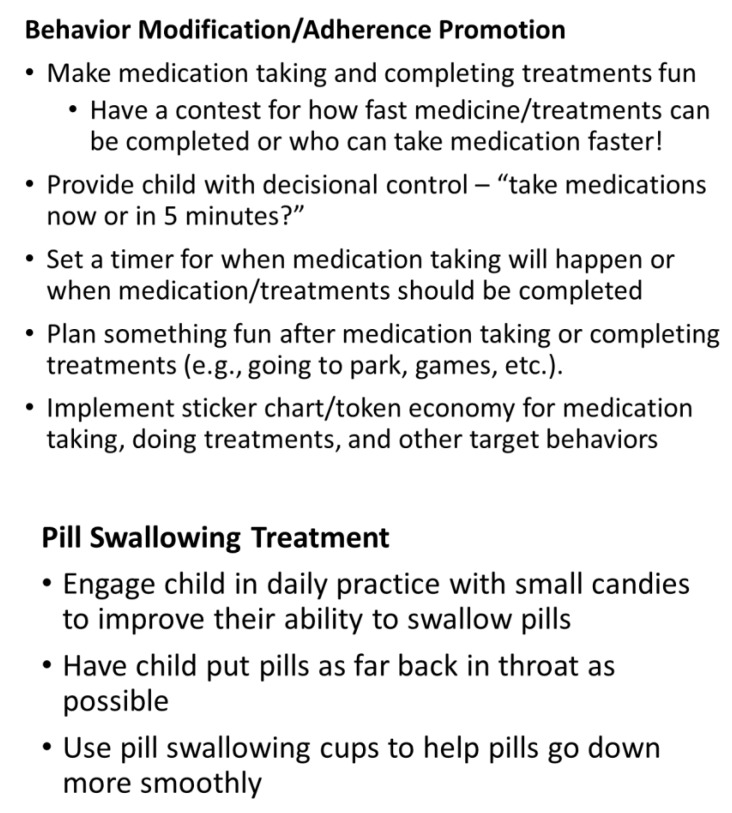
Strategies for improving medication adherence (Case 3).

As shown in Figure 3, a number of strategies were used to improve his adherence, including developing a daily routine and schedule that allowed the patient to check off the boxes when each treatment was finished and strategies for him to anticipate when during the day each treatment activity would occur, using strategies to improve the taste of medication, utilizing behavior modification and adherence promotion strategies to reduce fears and stress associated with mediation taking, and engaging the patient in pill swallowing treatment to maximize the success of taking medication. After several sessions, parents reported significant improvements in adherence and parent–child relationship dynamics (i.e., the patient was no longer resistant to taking medications).

### 1.4. Case 4. Medical Coping: Reducing Medical Trauma for a Pediatric Patient with a Urological Condition

The invasive medical procedures that many patients with chronic illness experience can be traumatizing to them, especially if they are viewed as scary or painful. A 9-year-old Dominican male with a complex medical history, including a urological disorder, required urodynamics studies (UDS) at regular intervals due to urinary retention. He was referred for pediatric psychology interventions to reduce medical trauma associated with UDS procedures and to improve his medical coping. He and his mother reported significant anxiety associated with “all” medical procedures and specific phobias related to all medical care, including attending non-invasive medical appointments for his complex medical issues. This young man also had a history of significant emotional and behavioral dysregulation during medical procedures in a high-anxiety setting (i.e., he would kick, cry, scream, push, hit, bite, and threaten medical providers). His dysregulation resulted in multiple occurrences of parents, medical providers, and security holding him down during procedures, which contributed to medical trauma and anxiety. 

**Figure 4:**
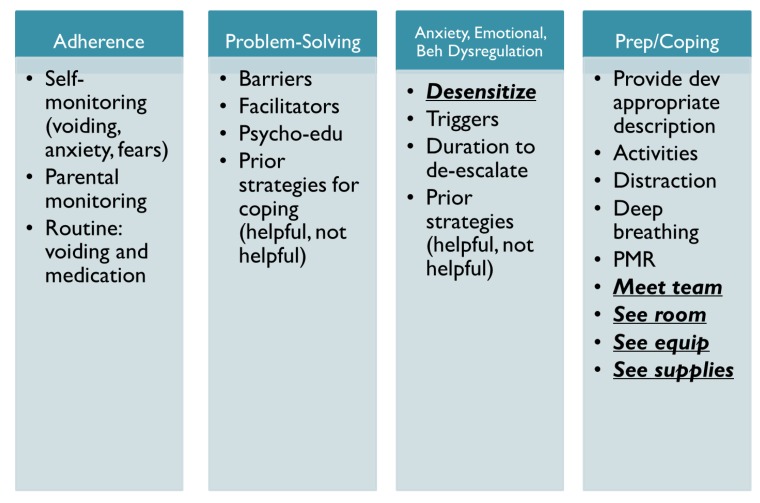
Medical coping and strategies to reduce medical trauma during medical interventions.

This patient was seen for two sessions by a pediatric psychologist to engage him in medical coping interventions and systemic desensitization (see Figure 4). Due to his urological disorder, this patient was required to void every two hours. Due to a history of pain during voiding, he would often wait to use the bathroom, which led to urinary retention and leakage issues. It was recommended that the mother and patient engage in adherence promotion strategies to enhance voiding, including scheduled toilet breaks, increased water during the day, and reminders to void. These strategies may ultimately lead to decreased frequency of UDS procedures. During both sessions, the psychologist engaged the family in guided problem-solving around strategies to improve adherence to voiding recommendations. We identified barriers to voiding success and implemented strategies to reduce barriers. The clinician also engaged the patient and mother in guided problem-solving around strategies to reduce fears associated with medical procedures with a focus on UDS procedures. The clinician provided psychoeducation regarding the procedure, including the equipment used during the procedure, allowed him to tour the exam room, and demonstrated how the various supplies would be used during the procedure. We identified effective coping strategies that he could use before, during, and after the procedure (e.g., distraction, guided imagery, deep breathing, progressive muscle relaxation). We also identified a calming activity that he could look forward to following the procedure. The day prior to the procedure, he met the team whom he would work with and we agreed that the psychologist would be present on the day of the procedure. With systemic desensitization interventions prior to the procedure, psychoeducation, and anticipatory guidance, this young man was able to successfully complete the procedure with no evidence of anxiety or behavioral/emotional dysregulation. Following the procedure, he expressed high confidence that he would be able to do the procedure again, especially since “it was not that bad.”

## 2. Psychological Interventions for Pediatric Chronic Illness: The Impact of Preventative and Therapeutic Interventions

As evidenced in the four case examples, pediatric psychologists provide comprehensive interventions for patients with acute and chronic medical conditions through the use of health promotion interventions, adherence and self-management promotion, cognitive behavioral therapy, behavioral therapy, medical coping, parent training, and motivational interviewing. As shown here, for the most impactful behavior change to occur, a combination of interventions is often the most effective. For all patients and families, significant gains and improvements were made in the target areas of treatment adherence, self-management, and medical coping. These gains and improvements were sustained over time as evidenced by discontinuation of treatment and no longer needing interventions. For all patients, treatment was discontinued when the patient and family demonstrated significant improvements in treatment goals. As shown here, pediatric psychologists have a diverse skill set and often treat a variety of presenting problems, including: promoting use of non-pharmacological strategies for pain management, coping with chronic pain, adjustment to medical disorders or life stressors, ongoing support with illness or hospitalizations, support with medical procedures, pill swallowing, needle phobia, and habit reversal training (i.e., an intervention technique used to replace negative behaviors with positive behaviors. This intervention is commonly used with PICA (a psychological disorder that often involves eating non-food items that do not have any nutritional value: e.g., hair, dirt, paint chips, Styrofoam), trichotillomania, intense scratching behaviors often associated with skin conditions, etc.). Pediatric psychologists also treat anxiety, mood concerns, attention and executive functioning concerns, neuro-developmental disorders, eating disorders, trauma, etc.

Pediatric psychologists are increasingly involved as key members of interdisciplinary research and clinical care teams in a wide range of pediatric and academic medical settings [11,19,23,24,25,26]. In fact, all four cases described in this article were seen as part of an interdisciplinary medical team that included psychology, social work, MDs, nurse practitioners, pharmacists, care partners, and educational consultants. Involving psychologists as key members of interdisciplinary collaborations is not only beneficial for patients and families, but also for other healthcare professionals. Psychologists bring a wealth of information and services to interdisciplinary teams, including: dissemination of evidence-based research into clinical practice, implementation of preventative and therapeutic interventions, delivery of brief interventions during medical visits, outpatient follow-up services for patients in need of more intensive services that cannot be delivered during routine medical visits, and long-term psychological treatment that can be delivered in tandem with medical care [11,19,23,24,25,26]. 

There are several interdisciplinary collaborative models of clinical care, which include integrated psychology models within medical sub-specialty clinics (i.e., having an integrated psychologist that provides direct clinical care services within the medical sub-specialty clinic); and models that utilize psychologists for consultation/liaison services only, but do not have an integrated psychology program as part of their medical service. There are benefits and drawbacks with each type of clinical model. Psychologists who are integrated team members often have the opportunity to provide continuity of care across the illness trajectory with the potential ability to see patients inpatient and outpatient (if needed). Models that utilize a consult/liaison approach may not be able to have continuity of care providers for repeat referrals; nor, will they be able to provide long-term services for patients who migrate between inpatient and outpatient settings.

### 2.1. Treatment Adherence and Coping with Chronic Illness 

The impact of chronic illness on pediatric patients and their families is multi-faceted. Patients who have a chronic illness may report physical limitations, which may subsequently impact their mobility, neuro-cognitive functioning, psychological functioning, social relationships, and development [3]. Furthermore, patients with a chronic illness may view themselves as different compared to healthy peers due to the significant lifestyle changes often involved with managing a chronic illness, including: increased healthcare utilization (e.g., high frequency of clinic visits, Emergency Department (ED) visits, and/or hospitalizations) and the need to take medications, monitor dietary intake, administer injections, check blood sugar, and/or use assistive devices (e.g., wheelchairs, walkers, etc.) [3]. 

Chronic illness management requires optimal adherence to prescribed treatment regimens. The field is moving toward the use of the term “adherence” to describe patient behavior rather than using the term “compliance” to ensure that there is an embedded framework of shared decision-making practices between healthcare providers and their patients [27,28,29]. The term “compliance” implies that patients are minimally involved in their healthcare decision-making and that the healthcare provider is the authority figure (i.e., “no matter what I say, you must comply”). In contrast, the term “adherence” demonstrates that the patient is an active participant in their healthcare, and they will have a shared responsibility for their health behaviors. Utilizing the term adherence ensures that there is an equal responsibility between patients and healthcare providers in disease management [27,28,29]. While adhering to complex treatment regimens will maximize disease management and ultimately health outcomes, these life-saving health behavior changes will likely also impact identity, social roles, psychological functioning, and general well-being [3]. Helping pediatric and young adult patients successfully navigate the complex world of managing a chronic illness will ultimately reduce and mitigate the risk of short-term and long-term health complications in a vulnerable patient population. 

### 2.2. Barriers and Facilitators to Treatment Adherence

It is well known that rates of nonadherence are high, which impacts both short-term and long-term health outcomes of pediatric patients with a chronic illness [10,19,24,25,26,30,31,32,33,34,35]. As shown in the case examples, patient-related factors that may contribute to nonadherence, include: neuro-cognitive and executive functioning deficits; forgetting to take medication; poor treatment-related knowledge; psychological factors like anxiety and depression; negative peer influences; dislike/distrust of authority including parental reminders to complete treatments; intentional nonadherence due to side effects or worries about specific medication; and developmental factors (e.g., transitioning from childhood to adolescence and adolescence to young adulthood). 

Psychological factors such as anxiety could serve as a protective factor. For example, increased worry about disease status and health outcomes can result in patients taking preventative actions to avoid adverse outcomes such as graft loss, transplant rejection, or death; as well as patients engaging in intensive planning around medication-taking behaviors to avoid missed doses [31,36]. On the other hand, anxiety may also serve as a risk factor, such as a patient with type 1 diabetes engaging in frequent blood sugar checking to avoid low or high blood sugars; or a patient with sickle cell disease perseverating on the taste and/or texture of medication and a fear of choking [31,36]. Additionally, a patient may be afraid to take their medication because of a fear of side effects or worry about how their illness will impact peer relationships [19,30,31,36]. Similarly, depression may contribute to higher rates of nonadherence. For example, a patient with depression may find treatments to not be worthwhile due to feelings of hopelessness and hence, will discontinue medication pre-maturely. Patients with depression are also had a greater risk of nonadherence (27%) compared to patients with anxiety (4%) [36]. 

As demonstrated in the first case, family-level (e.g., low socio-economic status, financial concerns, single-parent households, environmental concerns) can also pose a barrier to adherence, in addition to, increased psychosocial stress on the family system. With this case, multiple family members had a chronic illness and there were significant financial concerns. This may have contributed to this patient’s poor adherence to asthma medication if the grandmother had to choose between medication for another symptomatic child and him, especially if he was asymptomatic. Additionally, access to care may have been compromised due to transportation difficulties. Additionally, increased conflict between parent and child can result in intentional nonadherence due to adolescent rebellion and interpreting parental reminders as “nagging.” 

Previous research indicated that medication adherence was lower for adolescents who felt like there was minimal support at home and/or higher levels of family conflict between parent and child [36]. This finding was consistent across pediatric illness cohorts [36]. Similarly, increased rates of nonadherence could contribute to higher levels of family conflict [37,38,39,40]. For example, increases in family conflict and perceived parental criticism may interfere with productive problem-solving to reduce barriers ultimately leading to worse disease outcomes of pediatric patients [40]. Furthermore, by creating more adaptive adherence and self-management patterns in early childhood, patients will likely maintain these adaptive health behaviors over time, including during the transition from adolescence to young adulthood [19]. For example, child and adolescent emotion regulation may be influenced by their observations of how parents and other caregivers react to distressing events, including chronic illness management [19]. 

## 3. Evidence-Based Psychological Interventions to Promote Healthy Behaviors 

As shown here, comprehensive preventive and therapeutic health promotion interventions that are commonly used in clinical care can be tailored to individual patients and families and can be delivered in a systematic manner. The intervention models described here were tailored to the individual needs of each patient and participating caregiver and utilized tenets of health promotion and cognitive behavioral therapy [1,2,14,25,41,42,43,44], problem-solving, and motivational interviewing strategies [11,26,30]. This tailored and personalized intervention approach recognized that multiple stressors and demands could pose inevitable barriers to treatment success, including the development and/or maintenance of less than optimal health behavior patterns like poor adherence, less adaptive coping, and continued medical trauma. Moreover, patients’ psychological functioning and maladaptive problem-solving abilities could pose salient barriers to developing and refining more adaptive health behaviors, including anxiety, leading to increased risk of nonadherence patterns and/or poor coping, which ultimately impact health outcomes. 

The four clinical cases highlight the importance of integrating critical components of health promotion and cognitive behavioral therapy (CBT), including: consistent feedback about behaviors (e.g., patterns of medication adherence); identification of barriers affecting healthy lifestyles; psychoeducation about disease/treatment-related factors; strategies to improve psychological functioning and quality of life; and, promotion of healthy behaviors. CBT was found to significantly improve depression in patients with chronic diseases such as hemodialysis patients with depression [45]. In order to manage barriers to healthy lifestyle management, the intervention approaches tailored for each patient and family incorporated several relevant health promotion and CBT strategies. One of these was problem-solving theory, which is a major tenet of CBT and health promotion interventions. The clinician utilized problem-solving theory to teach and reinforce problem solving skills in individuals and families in an effort to enhance behavioral competence related to healthy lifestyle management. As part of this process, patients and families engaged in customized goal setting at the start of each intervention session. Consistent with problem-solving theory and motivational interviewing, the primary goals of the intervention process involved identifying “problems” that could be solved across the course of the intervention process and identifying barriers to achieving adaptive/optimal health behavior patterns. During each session, patients and families engaged in guided problem-solving exercises to identify barriers to healthy lifestyles by reviewing progress to goal attainment and reviewing objective patterns of medication adherence. As young people are receptive to health-related smartphone applications [46], online CBT can overcome geographical barriers [47] and offer a cost-effective solution [48].

With the four clinical cases, components of motivational interviewing were used to enhance development of sustained health behavior change. In addition, positive and adaptive parental behaviors, such as monitoring and modeling of constructive problem solving and encouragement, were shown to be important in interventions with children and AYA. For behavior changes to be sustained over time, it is vital to include both the patient and family as active participants in the intervention process. For all four cases, patients were the key participant in developing treatment goals and identifying barriers and facilitators to treatment success. Interventions would not have been successful if the patients were not the center of the intervention process with families being an equally active and vital part of treatment success. In all four cases, the voice of the child/adolescent resulted in identification of successful strategies for promoting patient and family success. These four cases highlight that when working with pediatric and AYA patients, it is critical that patients are provided an opportunity for one-on-one time with providers and that patients are given an active not passive voice in the therapeutic process. Pediatric patients are ultimately developing skills that will be vital for long-term success as an adult. Utilizing a patient- and family-centered intervention approach that focused on a discussion of problems that indirectly and directly influenced health outcomes ultimately resulted in positive health behavior changes that were sustained over time. Furthermore, the intervention model enhanced family and interpersonal relationships by promoting communication and collaboration between caregivers and patients.

## 4. Conclusions

Despite significant gains in survival rates for children, adolescents, and young adults diagnosed with a chronic illness, patients in this vulnerable developmental period are at an increased risk for developing one or more adverse effects related to their disease, treatment, or engaging in maladaptive health behaviors. Engaging in maladaptive health behaviors will ultimately increase the risk for developing adverse effects, including: increased rates of morbidity and mortality, impaired physical functioning, increased fatigue, obesity, comorbidities, increased psychological distress, and poor quality of life. With close attention including participation in preventive and therapeutic health promotion interventions, problematic health behaviors can be mitigated and ultimately prevented over time. It is well known that improved psychological functioning and adaptive coping can result in improved health status. The present paper provided four case examples illustrating techniques and principles of various psychological interventions that are often utilized when treating patients with a chronic illness. As evidenced in the four case examples, pediatric psychologists provide comprehensive interventions for patients with acute and chronic medical conditions through the use of multi-faceted health promotion interventions, which include adherence and self-management promotion, cognitive behavioral therapy, behavioral therapy, medical coping, parent training, and motivational interviewing. Our case series demonstrates that for the most impactful behavior change to occur, a combination of interventions is often the most effective. Furthermore, for treatment effects to be sustained over time, a combination of patient- and family-centered work is imperative.
